# Relationship between chlorine decay and temperature in the drinking water

**DOI:** 10.1016/j.mex.2020.101002

**Published:** 2020-07-22

**Authors:** Fernando García-Ávila, Carlos Sánchez-Alvarracín, Manuel Cadme-Galabay, Julio Conchado-Martínez, George García-Mera, César Zhindón-Arévalo

**Affiliations:** a Facultad de Ciencias Químicas, Universidad de Cuenca, Postal address 010107, Cuenca, Ecuador; b Unidad Académica de Salud y Bienestar, Universidad Católica de Cuenca, Sede Azogues. Postal address 030102, AzoguesEcuador; c Centro de Investigación, Innovación y Transferencia de Tecnología, Universidad Católica de Cuenca, Sede Azogues. Postal address 030102, Azogues, Ecuador; d Facultad de Ciencias Agropecuarias, Universidad Laica Eloy Alfaro de Manabí,Postal address 130803, Manta, Ecuador; e Universidad Nacional Agraria La Molina, Postal address 15024, Lima, Perú

**Keywords:** Bulk decay, Quality water, Drinking water network, Kinetic reaction order, Chlorine modeling

## Abstract

The bulk chlorine decay rate in drinking water supply systems depend on many factors, including temperature. In this document, the method to determine the order of reaction of chlorine with water is reported, as well as the method to estimate Kb (Bulk reaction rate constant). Experiments were carried out to determine the bulk chlorine decay, for which a set of water samples to determine the free residual chlorine every hour were analyzed. Chlorine concentrations were graphed against time and adjusted appropriately to the developed model. The experimental results showed that the average value of the mass decomposition rate was 0.15 h^−1^. It was shown that temperature affects the variation of the reaction rate of chlorine with water, Kb increases as temperature increases. In this manuscript it is reported:

• The method that allows determining the reaction kinetic order of chlorine with drinking water.
• The method that can help residual chlorine modelers in the correct definition of the bulk reaction rate constant.
• The effectiveness of the method for evaluating the decomposition of residual chlorine in drinking water distribution networks as a function of temperature.

## Graphical abstract

**Figure:**
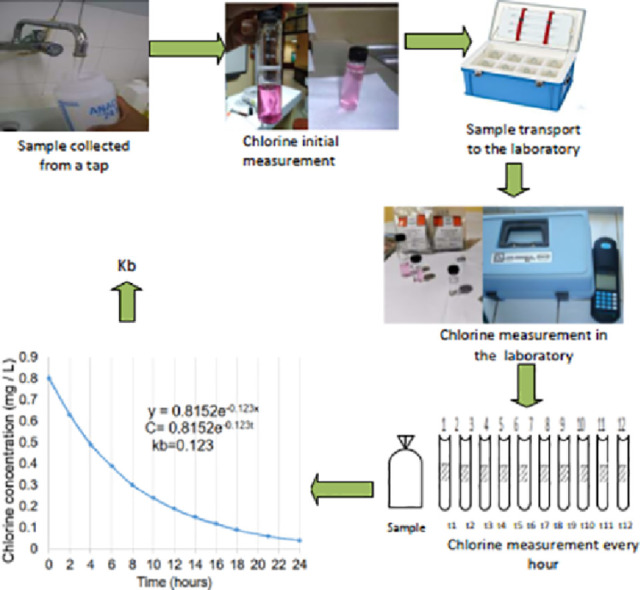

Specifications Table

**Table:** 

Subject Area:	Environmental Science
More specific subject area:	Drinking Water Quality
Method name:	Determination of the chlorine bulk decay rate
Name and reference of original method:	Powell, J.C., West, J.R., Hallam, N.B., Forster, C.F., Simms, J., 2000b. Performance of Various Kinetic Models for Chlorine Decay. J. Water Resour. Plan. Manag. 126, 13–20 [13]

## Method details

### Overview

To ensure the presence of residual chlorine in the Drinking Water Distribution Network (DWDN), several researchers have created a model for the chlorine bulk decay in drinking water [1,2]. This model allows evaluating chlorine levels in drinking water in the DWDN, allowing to predict the residual chlorine concentration over time, considering the chlorine initial concentration in the water treatment plant [3,4]. To model the decomposition of chlorine along its route within the distribution network, the reaction rate of chlorine with the mass of water (Kb) must be considered (chemical reactions of chlorine with the natural organic matter present in the water). Likewise, the reaction rate of chlorine with the wall of the pipeline where the water is transported (Kw) must be considered. [1,5]. Hua [6] in their studies, found that Kw represents only 10% of the reaction coefficient of chlorine with water (Kb). Temperature is one of the factors that mostly influence the chlorine bulk decay rate in DWDN [7], [8], [9]. The kinetics of the chlorine reaction with water is described by means of a first-order equation [2,[10], [11]:

$$C=C_{O}\;e^{-K_{b}t}$$

Where: C is the chlorine concentration at the time t; Co is the chlorine concentration at time zero; t is the time; Kb is the constant reaction rate, in h^−1^ or day^−1^.

Mathematical models of chlorine concentration in water distribution systems require that the chlorine bulk decay coefficient be quantified. This coefficient is a key parameter to model the behavior of residual chlorine in drinking water systems [12]. This suggests that these models could accurately predict the chlorine concentration in any part of the distribution network when a bulk decay belonging to the supply line understudy has been determined. Therefore, the objective of this study is to present the experimental methodology to determine the chlorine bulk decay, so that people who are entering this field of research, can easily find and replicate the value of Kb for the distribution network they are analyzing.

### Study area description

**Fig. 1:**
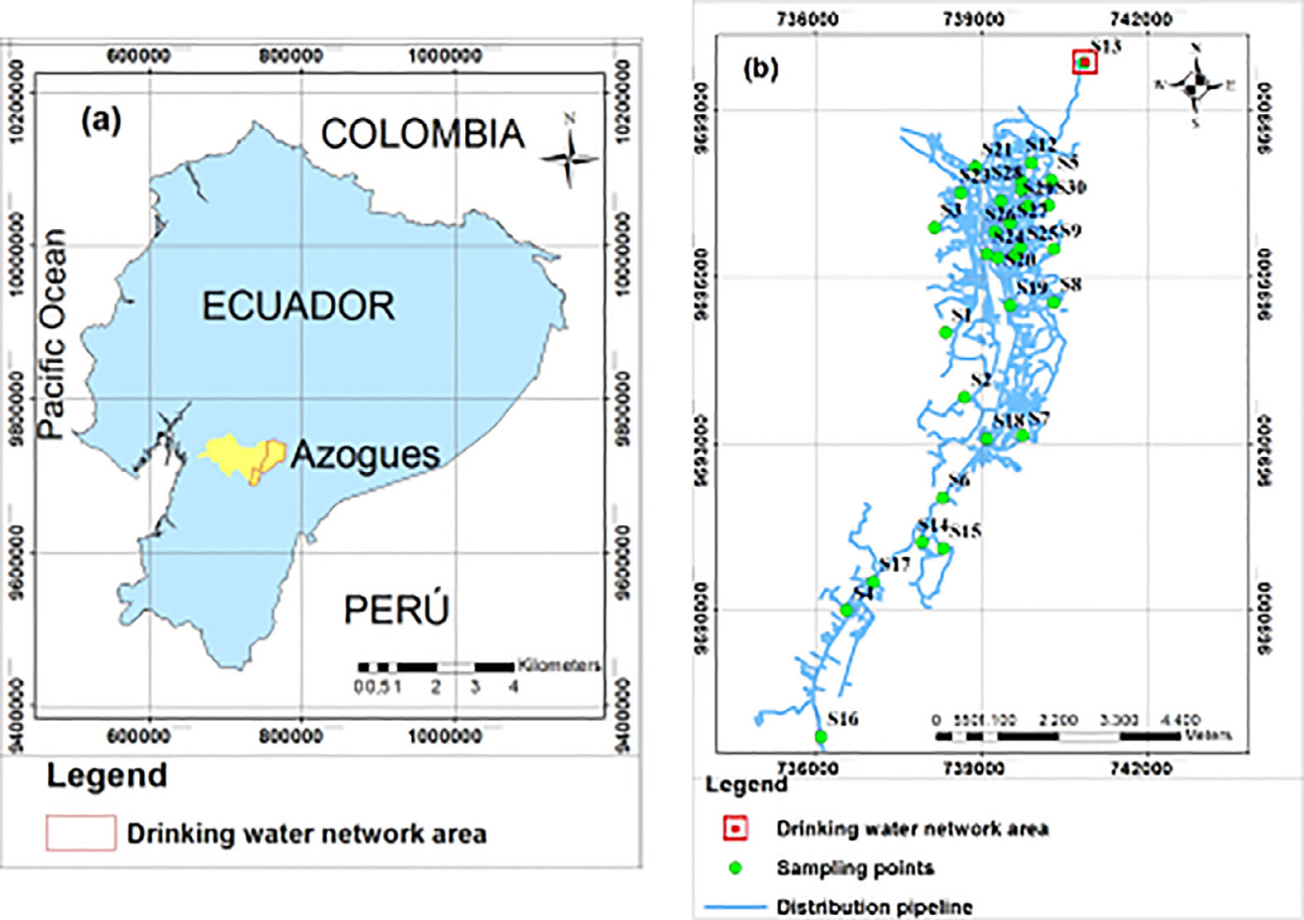
Location of the study area. (a) Location of the Azogues city, Republic of Ecuador (b) Location of the thirty sampling points of the drinking water distribution network where the chlorine decay constant was determined.

The treatment plant that supplies drinking water to the distribution network where this study was carried out is located in the Bayas parish, Azogues city, Ecuador, with its geographical coordinates are: latitude 2° 44′22″ S, longitude: 78° 50′54″ O. The supply network is made up of PVC pipelines with diameters between 32 and 315 mm, the total length of the supply line is 218.10 Km. The location of the distribution network is presented in Fig. 1.

### Sample collection and analytical procedures

Thirty samples were collected monthly for six months. The thirty sampling points were selected considering the length of the distribution network, the location of the reserve tanks and the number of users present in each of the six zones that make up the DWDN. Drinking water samples were obtained from household taps; commercial places like restaurants, workshops, car washes, shops; educational units; markets and distribution tanks.

The samples were collected in 1000 mL plastic bottles so as not to alter the samples for later analysis. The bottles were prepared previously, as recommended by the Ecuadorian standard and by studies carried out elsewhere [13]. The vessels were washed with concentrated calcium hypochlorite solution at 10 mg/L and allowed to stand for 24 h, then rinsed thoroughly with distilled water, and allowed to dry. To collect the sample from the taps, the sampling method recommended by the Ecuadorian standard INEN 2176 was followed [14]. The water was allowed to run for approximately two minutes to avoid collecting water that has been unused for a long period. The bottles were stored in a cooler to keep them at a constant temperature [15].

To determine the amount of free chlorine available in the collected water sample, the HACH DR 890 colorimeter was used, a DPD chemical powder (N, N‑diethyl-p-phenylenediamine) was added to the water sample. Chlorine in the sample as hypochlorous acid or hypochlorite ion (free chlorine or free available chlorine) immediately reacts with DPD indicator to form a magenta color which is proportional to the chlorine concentration[16]. This technique involves the addition of a reagent to a 10 mL water sample. All spot samples were taken in duplicate, the duplication of measurements reduced the error and provided a valuable quality control measure. Multiparametric HACH HQ40d was used to measure the pH and temperature. The first measurement of the residual chlorine concentration was made after taking the sample from the tap. Then the collected samples were stored in an incubator and transferred to the laboratory to continue with the measurement of residual chlorine every hour until the chlorine concentration value is close to zero. The monitoring results were necessary to determine the reaction order, as well as the constant Kb.

## Determining reaction order

With the results of the residual chlorine measurement, the order of kinetic reaction of chlorine with water was determined for each sample. To estimate the reaction order, the residual chlorine concentrations were graphed against their respective measurement time. If graphing the concentration of chlorine (C) against time gives a straight line, then the reaction is zero order. For a first-order relationship, Ln (C) was graphed against time, if the chosen order is correct, it tends to a straight line. For a second-order relationship, 1/(C) was graphed against time, if the chosen order is correct, the data tends to a straight line [17]. For each curve the value of the coefficient R^2^ was calculated, this determination coefficient allowed us to see what the precision of the correlation is, establishing the reaction order sought.

### Determination of bulk decay coefficient Kb

To obtain the reaction constant of chlorine with the mass of water, the procedure suggested by [13,[18], [19] was used. The following steps carried out in order were:

1. Collect water samples by storing them in clean one-liter bottles, this volume is due, to the high number of residual chlorine measurements per bottle over time.
2. Measure the concentration of residual chlorine once the sample has been taken, recording the time that started the measurement.
3. The samples collected at the different points of the distribution network were stored in an incubator and transferred to the laboratory in an isolated box to continue with the measurement of residual chlorine.
4. In the laboratory, the chlorine concentration of the samples' water was measured at constant time intervals (every hour), until the chlorine concentration value was close to zero.
5. With the above, the chlorine decay was obtained in relation to the reaction of the chlorine with the water body only (the reaction of the water with the pipeline wall is excluded).
6. The data obtained from the measurements were processed through a curve fitting program, such as Excel, and an exponential decay curve was constructed.
7. The coefficient Kb was obtained from the equation: C = C_O_ e^−Kbt^, after making the respective exponential adjustment (Fig. 2).

**Fig. 2:**
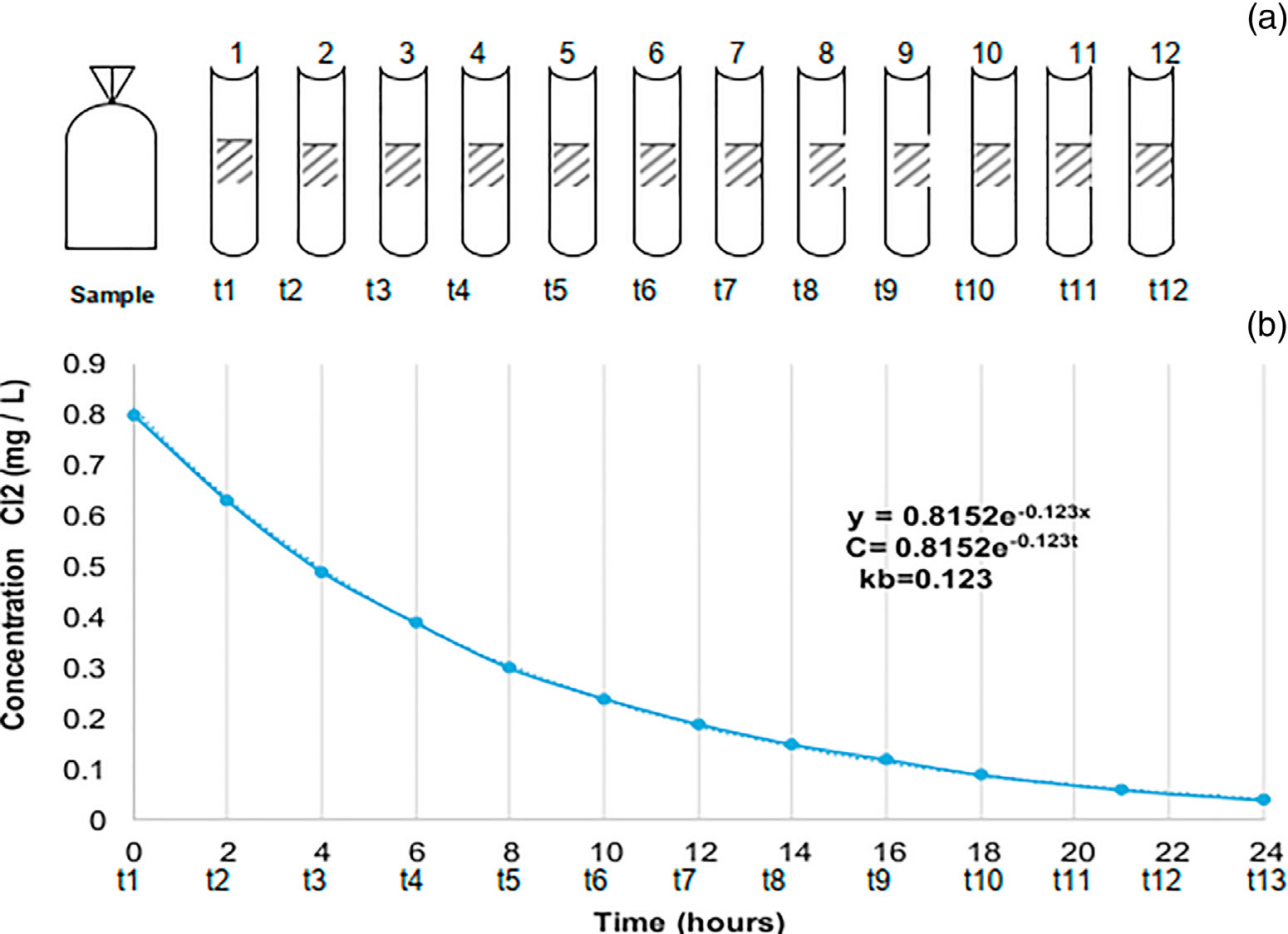
Laboratory Bulk Decay Coefficient Determination Diagram: (a) Bulk Decay Bottle Tests, (b) First order exponential fit for the equation.
8. To obtain a representative average constant of chlorine reaction with water, an average was made with the Kb values of each sample.

## Method validation

### Kinetic reaction order

**Fig. 3:**
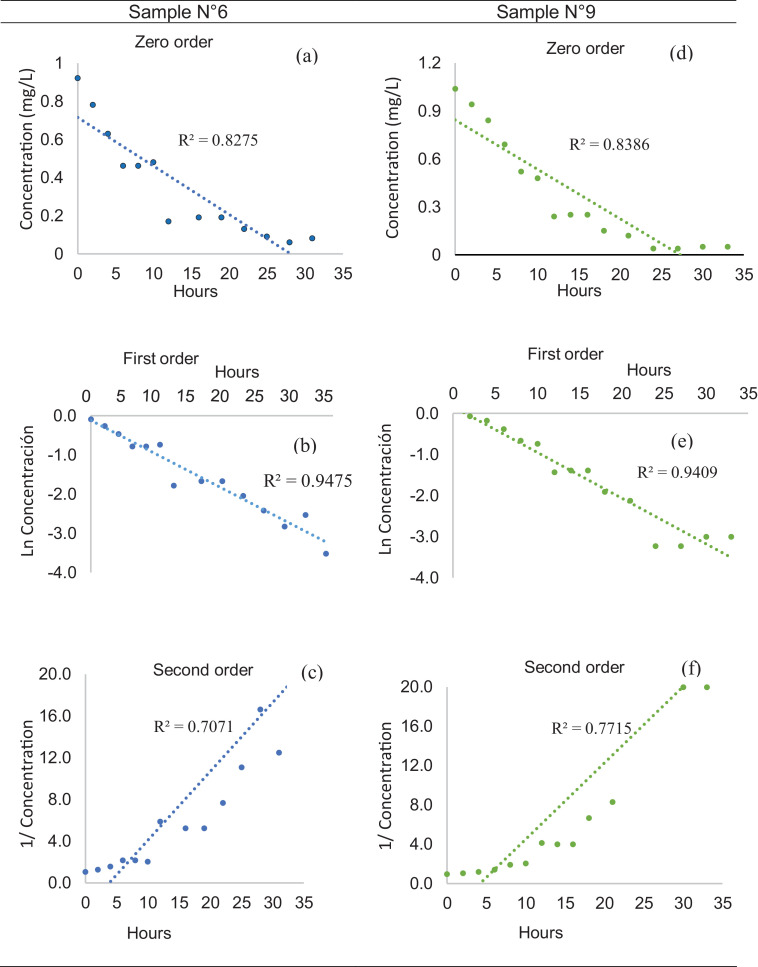
Determination of the reaction order. (a) and (d) Graphs of chlorine concentration as a function of time; (b) and (e) Semi-log graphs of chlorine concentration as a function of time; (c) and (f) Graphs of inverse concentration of chlorine as a function of time. Sample No. 6 (a) Zero-order, (b) first-order, (c) second-order; Sample No. 9 (d) Zero-order, (e) first-order, (f) second-order.

After having carried out the methodology described above, an example of how the reaction order was determined is presented in Fig. 3. In this figure, the application for sample No. 6 of the month of August is presented. When the residual chlorine concentration (C) was plotted against time for a zero-order reaction, an R² value of 0.8275 was obtained (Fig. 3a). By plotting Ln (C) against time for a first-order reaction, an R² of 0.9475 was obtained (Fig. 3b). Meanwhile, when 1/(C) was plotted against time for a second-order reaction, an R² of 0.7071 was obtained (Fig. 3c). Therefore, it is considered that there is an order one reaction because a better R² was obtained.

$$C=C_{O}\;e^{-K_{b}t}.$$

In the same Fig. 3, another example is presented, in this case for sample No. 9; an adjustment coefficient R² of 0.8386 was obtained considering a zero-order reaction (Fig. 3d); an R² of 0.9409 considering an order one reaction (Fig. 3e) and an R² of 0.7715 considering an order two reaction (Fig. 3f). Therefore a first-order is ratified. The same procedure was followed for all the samples during the six months of monitoring, the results confirmed that for the present study there is a first-order reaction.

### Obtaining of the chlorine bulk decay coefficient

**Fig. 4:**
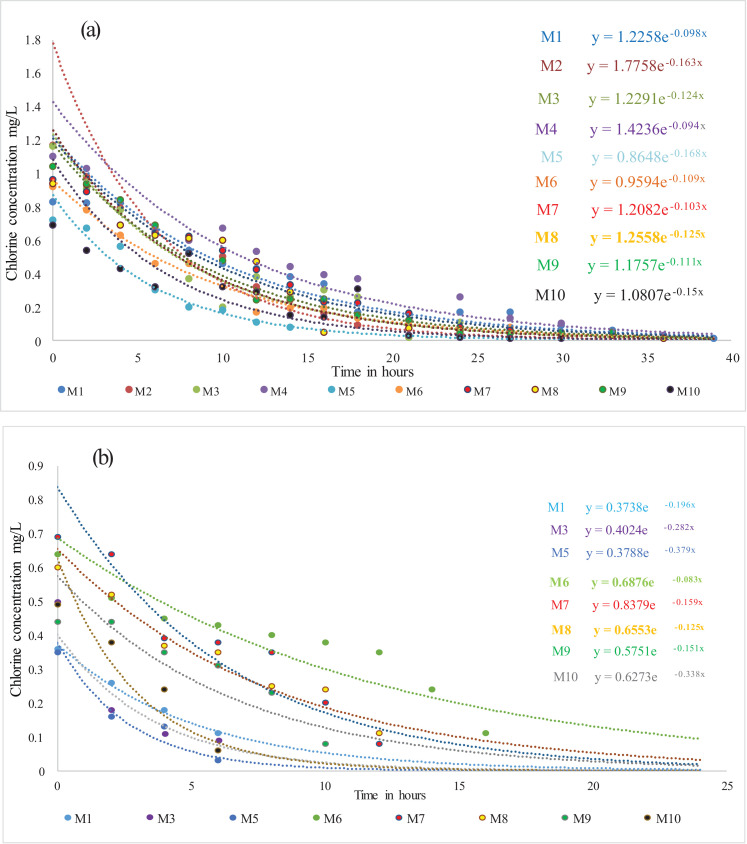
Obtaining the chlorine bulk decay coefficient; for ten sampling points in July (Fig. 4a); for ten sampling points in August (Fig. 4b).

After it was determined that the reaction order was first-order, the measured chlorine concentrations were graphed as a function of time, an exponential adjustment was made to obtain the reaction coefficient Kb for each sample. As an example, Fig. 4 presents the obtaining of Kb for ten sampling points for the month of July (Fig. 4a) and eight points for the month of August (Fig. 4b). In the equations presented in Fig. 4a, it is observed that the mass decay coefficient (Kb) is equal to 0.098 h^−1^ in sample 1 (M1); 0.163 h^−1^ in sample 2 (M2); 0.124 h^−1^ in sample 3 (M3); 0.094 h^−1^ in sample 4 (M4); 0.168 h^−1^ for sample 5 (M5) of the month of July. Following this procedure, the Kb values ​​were determined for all the samples of the six months monitored. The negative sign presented in the equation refers to the reduction of chlorine over time.

**Fig. 5:**
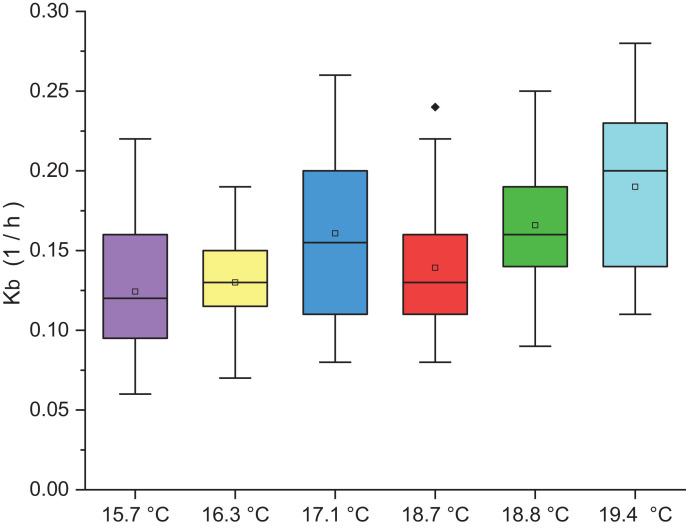
Variation of the decay constant Kb as a function of the monthly average temperature.

The experimental results of the determination of the bulk decay constant (Kb) as a function of the monthly average temperature are presented in Fig. 5. The temperature observed during the measurements of residual chlorine affected the bulk decay constant of the chlorine. The temperature of 15.7, 16.3, 17.1, 18.7, 18.8 and 19.4 °C corresponds to the months of July, August, September, January, February and March respectively. In this figure it can be seen that as the temperature increases, the coefficient Kb increases, that is, there is a greater residual chlorine decay with the increase in temperature. Kb values of 0.124; 0.163; 0.128; 0.133; 0.192; 0.163 h^−1^ for July, August, September, January, February and March respectively were obtained. For this study, a Kb average of 0.154 h^−1^ was obtained. These obtained Kb results can be used for modeling residual chlorine in a DWDN.

The monitored points that are close to the treatment plant had residual chlorine values between 0.6 and 0.8 mg/L, while the furthest points were between 0.21 - 0.3 mg/L. The first monitoring point was precisely in the distribution tank of the treatment plant, where values between 0.80–0.89 mg/L were obtained. Great variation was found in the residual chlorine values, at the monitoring points, where the highest value was 0.89 mg/L (tank), while the lowest value was 0. 21 mg/L (network termination). The average chlorine initial dose in the water distribution network of the study area was 0.69 mg/L, which is small since the upper limit of the Ecuadorian standard is 1.5 mg/L [20], but it is higher than the limit of WHO that suggests 0.5 mg/L [21]. The water quality parameters in this study area have values within the limits of the WHO standards [21], with an average turbidity of 0.51 NTU, which means that this water has a good quality [22].

An average value of the chlorine bulk decay constant of 0.154 h^−1^ was obtained. This value is higher than the Kb value of 0.0229 h^−1^ reported by Rossman [1] and other studies. This variation is due to the fact that the reaction of chlorine with water depends on the particular conditions of each zone, such as temperature, organic matter content, operation and maintenance of the distribution network. The chlorine decay is proportional to the content of organic matter in the water (expressed by dissolved organic carbon). With a higher content of organic matter, a faster decay occurs.

The practice of chlorination in the treatment plant in the study area was not adequately regulated, which resulted in different chlorine initial concentrations at the monitored points. High levels of residual chlorine show excessive chlorine doses that can result in high operating costs for service providers, as well as potential health problems for consumers.

In the supply network under study, the best way to ensure that there is always residual chlorine amount in the most distant points to the treatment plant is to dose a quantity of disinfectant in the plant, in such a way that it allows obtaining a concentration 0.8 - 0.9 mg/L at the outlet of the plant, and a concentration of 0.2 - 0.3 mg/L at these remote points, such as presented in the points S16, S17 (Fig. 1b). If the dosage is reduced in the treatment plant, it is not possible to obtain residual chlorine in the furthest points. In some networks that are too long, it can be difficult to maintain the proper residual chlorine amount at all points. Therefore, in this case, it is recommended to carry out a study that allows analyzing the need to fractionate the chlorine dosage by installing chlorinators at various points in the network.

The results obtained in this study validate the practicality of the proposed methodology for the conditions given in developing countries, however, the chlorine decomposition coefficients must be calibrated for the conditions of the distribution network; the ideal would be to determine the chlorine bulk decay of the study site.

## Declaration of Competing Interest

The authors declare that they have no known competing financial interests or personal relationships that could have appeared to influence the work reported in this paper.
